# Psychometric properties of the English and Hindi versions of the Brief Inventory of Thriving for use among Indian adolescents

**DOI:** 10.1038/s41598-024-83078-z

**Published:** 2024-12-30

**Authors:** Usama Ghayas Syed, Shikha Dixit, Margaret L. Kern

**Affiliations:** 1 Department of Humanities and Social Sciences, Indian Institute of Technology Kanpur, Room No. FB-613, Faculty Building, 6th Floor, Kalyanpur, Uttar Pradesh 208016 India; 2 Indian Institute of Technology Kanpur, FB-153, Kalyanpur, Uttar Pradesh 208016 India; 3 Faculty of Education, Centre for Wellbeing Science, The University of Melbourne, Level 2, 100 Leicester Street, Carlton, VIC 3010 Australia

**Keywords:** Brief Inventory of Thriving, Adolescents, Measurement invariance, Language equivalency, Psychometric testing, Psychology, Human behaviour

## Abstract

The Brief Inventory of Thriving (BIT) provides a holistic measure of well-being, but has only been validated for adults, and does not have a Hindi version. The present study investigated the unidimensional structure, internal consistency, convergent/discriminant, and criterion validity of both the original English version of the BIT (BIT-E) and its Hindi-translated version (BIT-H) among adolescents in India. Further, we tested measurement invariance across these two language versions, gender, and academic disciplines. A total of 534 adolescents were recruited across two samples (*N*_*1*_ = 224 and *N*_2_ = 310) from five schools using convenience sampling. Both versions demonstrated excellent psychometric properties, with unidimensional structure, good internal consistency, convergent/discriminant, and criterion validity with a number of psycho-educational correlates. Partial scalar invariance was achieved across language versions and gender, while strict invariance was established across academic disciplines. The BIT, in both English and Hindi, appears to be an excellent measure of well-being for adolescents. Limitations, directions for future research, and recommendations for using the BIT-E and BIT-H among adolescents in research and applied settings are discussed.

## Introduction

With some exceptions, across the 20th century, researchers and practitioners primarily approached human behavior from a deficit perspective, focused on mental illness rather than well-being and thriving^1^. This bias also extends to adolescents, a period of developmental changes characterized by physiological, biological, social, emotional, cognitive, and behavioral adjustments^2^. Considering the high rates of mental illness, self-harm, eating disorders, and externalizing behavior among adolescents^3^, the deficit focus is helpful. However, the lack of mental illness does not necessarily equate to well-being in life^2^. To protect young people from developing mental illness or at least to lessen the severity, supporting and enhancing the well-being of adolescents has increasingly become a goal of educators, families, and policymakers. In line with this goal, researchers and mental health professionals have shown greater interest in understanding well-being and its influence on positive youth development^2^. Adolescents with a higher level of well-being demonstrated greater academic success^4^, higher self-esteem^5^, better health in adulthood^6^, and lower metabolic syndrome risk^7^, among other positive outcomes^2^.

Here we focus on subjective well-being, acknowledging that objective well-being is important for creating the conditions within which subjective well-being can be experienced^8^. While there are numerous definitions, models, frameworks, and theories of subjective well-being, well-being consistently reflects feeling good and functioning well across multiple life domains (e.g., physical, mental, emotional, cognitive, spiritual, and financial domains^9,10^. Feeling good refers to hedonic well-being, which focuses on maximizing pleasure and minimizing pain^11^. Functioning well refers to eudaimonic well-being, which focuses on the life well-lived^11^. Hedonic and eudaimonic dimensions are highly correlated^12,13^, yet are still distinctive facets, with current recommendations being that both facets should be included in well-being measures^14,15^.

Considering the need to measure what matters^16^, psychometrically valid measures of well-being have been developed. This includes the Life Satisfaction scale^17^, the Psychological Well-being Scale^18^, the PERMA-Profiler^19^, and the Comprehensive Inventory of Thriving^20^, among many others. Some measures of adolescent well-being exist^21^, but existing measures such as the EPOCH^22^ measure of adolescent well-being focus on psychological functioning rather than well-being per se, are lengthy in nature (which can undermine validity of the measure due to limited attention spans by participants), have not been translated across languages, are limited to use with Western, Educated, Industrialized, Rich, and Democratic (WEIRD) cultures^23^, and have been validated with adults rather than adolescents. The Brief Inventory of Thriving (BIT)^20^ potentially can address these limitations, as it directly focuses on psychological well-being and is brief in nature (10 items). However, it has not been translated to Hindi, has primarily been used in WEIRD cultures, and has been validated with adults rather than adolescents. To test its suitability with Indian adolescents, we translated the measure into Hindi and test the psychometrics of the English and Hindi versions of the measure with two groups of Indian adolescents.

To develop the BIT, the authors combined prominent theories of well-being to select seven key dimensions of well-being, “(1) subjective well-being; (2) supportive and enriching relationships; (3) interest and engagement in daily activities; (4) meaning and purpose in life; (5) a sense of mastery and accomplishment; (6) feelings of control and autonomy; and (7) optimism” (p. 252)^20^. Using these seven dimensions, they created the 54-item Comprehensive Inventory of Thriving (CIT) and its shorter version, the Brief Inventory of Thriving (BIT). Items demonstrating the highest factor loadings from the sub-dimensions of the CIT were selected to construct the BIT^20^. The BIT has one item each for meaning, positive emotions, life satisfaction, accomplishment, support, self-worth, optimism, self-efficacy, belonging, and engagement. After the development and initial validation of the BIT in the United States^20^, it has been validated with adult populations across various countries. Studies conducted in Germany^24^, Brazil^25^, Italy^26^, Turkey^27^, and China^28^ found the BIT to be a psychometrically sound instrument for adults. A study testing the BIT’s factor structure and cross-cultural invariance across ten nations, including the US, Australia, Germany, Mexico, Russia, Spain, Turkey, China, Singapore, and India^29^, confirmed its unidimensional structure and found it a culturally invariant instrument across the studied nations. Sorgente and colleagues tested the gender (male vs. female), age (18–24 vs. 25–30), and cross-cultural invariance of the BIT across Chinese, Italian, and Portuguese adult populations and found evidence of invariance^30^.

Overall, the BIT appears to be a reliable and valid instrument of holistic well-being with adult populations in WEIRD as well as non-WEIRD nations. However, no study has examined the BIT’s psychometric properties among adolescents, indicating the need to investigate and validate BIT’s psychometric properties among this age group. Moreover, the measure has not been translated to Hindi, limiting its use with Indian populations. Therefore, we developed the Hindi version of the BIT and tested its equivalency with the original English version using measurement invariance testing among Indian adolescents.

## The present study: aims and hypotheses

The present study had three primary aims. First, we aimed to translate the BIT into Hindi and evaluate the factor structure of both the original English (BIT-E) and the Hindi-translated (BIT-H) versions of the BIT among Indian adolescents. We used the rigorous methodological approach by Sousa and Rojjanasrirat in translating and adapting the BIT into Hindi^31^. Since previous studies have consistently replicated the unidimensional structure of the BIT among adults, we hypothesized that the unidimensional structure would be confirmed for both the BIT-E and BIT-H among Indian adolescents.

While most translated versions of the BIT have successfully replicated its unidimensional structure, they may function differently across demographic groups^32^. Thus, our second aim was to test measurement invariance (MI) across the two language versions, gender, and academic disciplines. Establishing MI is a crucial step in psychometric validation to ensure the scale measures the same construct across diverse groups^33^. MI tests the psychometric equivalence of the construct by increasingly constraining the factor structure (configural invariance), factor loadings (metric invariance), intercepts (scalar invariance), and residual variances (strict invariance). Given the linguistic diversity among Indian adolescents, as Hindi and English are commonly used languages in educational and social contexts, testing MI across languages was necessary to confirm that the Hindi-translated BIT is equivalent to the original, allowing valid cross-language comparisons. Further, examining MI across gender helps ensure that the BIT measures well-being consistently for both boys and girls, reducing the risk of gender bias. Similarly, testing invariance across academic disciplines accounts for potential differences in students’ well-being perceptions due to the distinct experiences associated with their field of study. Demonstrating MI across these groups enhances the BIT’s applicability and ensures its reliability in diverse educational contexts. Based on the previous findings, we hypothesized that invariance would hold across language versions, gender, and academic disciplines.

Third, we assessed the reliability, convergent/discriminant, and criterion validity of the BIT-E and BIT-H. We hypothesized that both the BIT-E and BIT-H would demonstrate good reliability, as well as strong convergent, discriminant, and criterion-related validity, given that previous studies have shown robust psychometric properties across various cultural contexts. We used different sets of measures to establish the validity of the BIT-E and BIT-H to reflect the distinct contexts in which these versions were administered. For the BIT-E, we assessed convergent/discriminant validity by examining correlations with life satisfaction, positive affect, negative affect, subjective happiness, and student satisfaction. These measures are well-established in the well-being literature and align with the previous validation studies^20,28^ of the BIT. For the BIT-H, we assessed both convergent/discriminant validity and criterion-related validity. For convergent/discriminant validity, we examined correlation with student satisfaction, which was also used in the BIT-E, as it reflects a key aspect of well-being in the educational context. For criterion-related validity, we included measures related to school outcomes such as generic skills in school, academic self-efficacy, student engagement, and perseverance. These constructs were selected because they are particularly relevant to the academic and developmental experiences of Hindi-speaking students, reflecting key aspects of both academic and psychosocial functioning.

## Method

### Participants

A total of 534 adolescents studying in the 11th grade were recruited across two samples using a convenience sampling approach from five government schools affiliated with the Central Board of Secondary Education (CBSE) located in the Hindi-speaking region of India. All the participants were from the Kanpur district (an urban area), and their medium of instruction was English. For Sample 1, 250 questionnaires in their original English versions were distributed among adolescents in two schools, against which 224 (response rate = 89.60%) adolescents (boys = 116; *M*_age_ = 16.09 years; *SD* = 0.82; range = 15–18: girls = 108; *M*_age_ = 16.05 years; *SD* = 0.73; range = 15–18) returned the questionnaire. There were 133 adolescents from the discipline of science, whereas 91 were from commerce. For Sample 2, 470 questionnaires in their Hindi-translated versions were distributed in three schools, and 323 (response rate = 68.72%) adolescents returned the questionnaire. Out of 323 questionnaires, we removed the data of 13 participants due to many missing values or failing in more than two control questions. These control questions were forced-choice questions in which participants had to select the pre-specified responses (e.g., “*Please select strongly agree as the response to this item”* or *“Please select strongly disagree as the response to this item*”) to check whether the participants were attentive to the items while giving their responses. Therefore, the data from 310 adolescents (boys = 163; *M*_age_ = 16.09 years; *SD* = 0.70; range = 15–18, *N* = 6, who did not indicate their age: girls = 147; *M*_age_ = 16.10 years; *SD* = 0.59; range = 14–18, *N* = 4, who did not indicate their age) were used in the present study. These adolescents were from the disciplines of science (*N* = 225), commerce (*N* = 54), and the humanities (*N* = 31).

### Procedure

After receiving approval from the Institutional Ethics Committee, the first author secured permission from the principals of the selected secondary schools. Participants received a packet of questionnaires, including a formal letter introducing the study and requesting participation. The questionnaire package included some demographic questions and scales in their original English version for Sample 1 and their Hindi version for Sample 2. Demographic data included participants’ gender, self-reported age, and chosen discipline. For Sample 1, the package contained the BIT-E along with the Concise Measure of Subjective Well-being (COMOSWB), the Subjective Happiness Scale (SHS), and the Student Satisfaction Scale (SSS). For Sample 2, the package contained the BIT-H, Generic Skills Scale (GSS), Academic Self-efficacy Scale (ASES), the Student Satisfaction Scale (SSS), and two subscales of Student Engagement (SE) and Student Perseverance (SP) from the EPOCH model of well-being of adolescents^22^.

Participation in the study was voluntary, and participants received no compensation. The participants were assured about the anonymity and confidentiality of their data and the use of the data only for research purposes. Participants were free to withdraw from the study during or after the data collection. All participants signed an informed consent form. Data were collected in the given time slots, and during the data collection, classroom teachers were absent to assure participants about the confidentiality of their responses.

### Measures

#### Translation and adaptation

All the scales used in Sample 2, including the BIT, were translated and adapted into Hindi following a comprehensive five-step process developed by Sousa and Rojjanasrirat^31^. First, two independent bilingual translators translated all the scales into Hindi. Second, the first author compared the two translated versions to rule out any ambiguity or discrepancy in words, sentences, or their meaning. Differences between the translations were discussed, and a consensus was reached to create a unified preliminary Hindi version of the scales. In the third step, another independent bilingual translator, who had not seen the original English version of the scales, back-translated the synthesized Hindi versions into English. This step was conducted to ensure that the translated items retained the original meaning and intent of the scale. In the fourth step, two experts compared the relevance, format, words, similarity in meaning, and grammatical structure of the back-translated versions of the scales with the original scales. Any remaining discrepancies were resolved by the experts. This way, a pre-final version of all the scales used was approved for pilot testing. In the fifth step, the pre-final version of all the scales were evaluated by 18 adolescents (boys = 8, girls = 10) through an online pilot study. Their ages ranged from 14 to 18 years (*M*_age_ = 15.77 years; *SD* = 0.85). The sample included students from the Science (*n* = 15) and Commerce (*n* = 3) streams. The participants were asked to evaluate the clarity of each item using a dichotomous scale (i.e., clear vs. unclear). Items were retained if at least 80% of the participants rated them as clear^34^. All the BIT-H items were evaluated as clear; however, two items related to other scales did not reach the criteria. Based on the participants’ feedback, we made minor changes to those two items, which resulted in the final translated measures.

#### The Brief Inventory of Thriving

The 10-item Original English and Hindi-translated versions of the Brief Inventory of Thriving (BIT) were used to measure adolescents’ well-being^20^. All items are positively worded, with a higher score on the scale indicating a greater level of well-being. A sample item included *“I feel good most of the time.”* Participants responded on a 5-point scale, where 1 indicated *strongly disagree* and 5 indicated *strongly agree*. The reliability and validity of both versions of the BIT are reported in the Results Section.

#### The Concise Measure of Subjective Well-Being

The Concise Measure of Subjective Well-being (COMOSWB) was used to measure subjective well-being^35^. With nine items, it assesses cognitive (i.e., life satisfaction) and affective (i.e., positive and negative affect) domains of subjective well-being. Each domain has three items. For the cognitive domain, participants indicated their agreement or disagreement on a 7-point scale (1 = *strongly disagree*; 7 = *strongly agree*); for instance, *“I am satisfied with the personal aspects of my life.”* For the affective domain, participants were asked to indicate the frequency of the positive emotions (e.g., *joyful*) and negative emotions (e.g., *irritated*) they experienced in the past month on a 7-point scale (1 = *never*; 7 = *always*).

#### Subjective Happiness Scale

We used the 4-item Subjective Happiness Scale (SHS) to assess participants’ happiness^36^. The measure gives a broader picture of well-being than instruments that look at happiness more narrowly. Through two items, respondents rated their happiness in absolute and relative terms. The scale is a 7-point scale, such as *“in general*,* I consider myself…”*; rated from 1 (*not a very happy person)* to 7 (*a very happy person).* The other two items characterize happy and unhappy people and ask the respondents to rate (1 *= not at all* and 7 *= a great deal*) the extent to which these characteristics describe them. A sample item is *“Some people are generally very happy. They enjoy life regardless of what is going on*,* getting the most out of everything. To what extent does* this characterization describe you?”

#### Student Satisfaction Scale

The 7-item Student Satisfaction Scale (SSS) was used to assess student satisfaction with school^37^. The original scale measures college satisfaction. We modified certain words and phrases to adapt them for school students. The SSS prompts the respondents with the phrase *“Please indicate your satisfaction or dissatisfaction in terms of”* and asks them to rate on a 7-point scale from 1 (*very dissatisfied*) to 7 (*very satisfied*), such as *“the quality of teachers you have in your school.”*

#### Generic Skills Scale

The Generic Skills Scale (GSS), a 6-item subscale of the course experience questionnaire, was used to assess the generic skills of the participants^38^. The GSS prompts the respondents with the phrase *“The courses taught in school”* and asks them to rate on a 7-point scale from 1 (*strongly disagree*) to 7 (*strongly agree*), such as *“developed my problem-solving skills.”*

#### Academic Self-efficacy

The seven items Academic Self-efficacy Scale (ASS) was used to assess the academic self-efficacy^39^. Participants rated their agreement or disagreement with the items using a 7-point scale, where 1 indicated *strongly disagree*, and 7 indicated *strongly agree*. A sample item is *“I know how to study to perform well on tests.”*

#### Student engagement and perseverance

Student engagement and perseverance were measured using the engagement and perseverance subscales of the EPOCH measure of adolescent well-being^22^. Both subscales have four items. Certain words and phrases were modified and adapted for school-going adolescents. Sample items include *“I get completely absorbed in school-related activities”* and *“I finish whatever school activity I begin”* for the engagement and perseverance subscales, respectively.

### Data analyses

#### Ancillary data analyses

First, we checked the data for missing values, which were below 2% for both samples (Sample 1; 1.071%: Sample 2; 0.365%). Little’s test indicated that the data were missing completely at random (MCAR) for both samples (Sample 1; χ^2^(551) = 542.276, *p* = .596: Sample 2; χ^2^ (762) = 794.640, *p* = .200). Since all the scales used a 5-7-point Likert scale, we replaced the missing values with the median values on the scale. After that, we used the imputed datasets in the subsequent analyses. Further, we tested the assumptions of univariate and multivariate normality by using the WebPower online calculator (retrieved from: https://webpower.psychstat.org/models/kurtosis/), developed by Zhang and Yuan^40^. We tested the assumptions of univariate normality based on Finney and DiStefano’s criteria for both samples^41^. The skewness and kurtosis values should not exceed ± 2 and ± 7, respectively, to determine the absence of violations of univariate normality. We also tested the assumption of multivariate normality. The value of Mardia’s multivariate kurtosis should be less than 5^42^. The data indicated that all the items of the BIT for both samples were univariate normal. In Sample 1, skewness values ranged from − 1.388 to 0.067, while in Sample 2, they ranged from − 1.730 to -0.481. Kurtosis values ranged from − 0.665 to 1.906 in Sample 1 and from − 0.486 to 3.101 in Sample 2. However, Mardia’s multivariate kurtosis indicated a violation of the assumption of multivariate normality (Sample 1; Multivariate Kurtosis (C.R.) = 6.52: Sample 2; Multivariate Kurtosis (C.R.) = 24.12. Table 1 indicates the item-level descriptive statistics for the BIT-E and BIT-H.

**Table 1:** Item-level descriptive statistics, item-total, and inter-item correlations for the BIT-E and BIT-H.

Item	M	SD	Sk	Ku	ITC	1	2	3	4	5	6	7	8	9	10
1. My life has a clear sense of purpose. 1. मेरे जीवन का उद्देश्य स्पष्ट है।	3.82 (4.10)	0.76 (1.10)	-0.338 (-1.277)	-0.197 (1.017)	0.489 (0.500)	1	0.44	0.29	0.21	0.28	0.38	0.35	0.38	0.22	0.28
2. I am optimistic about my future. 2. मैं अपने भविष्य को लेकर आशावादी हूँ।	4.01 (4.13)	0.81 (1.02)	-0.426 (-1.283)	-0.427 (1.301)	0.482 (0.445)	0.43	1	0.26	0.18	0.27	0.39	0.26	0.28	0.21	0.25
3. My life is going well. 3. मेरा जीवन अच्छा चल रहा है।	3.66 (3.73)	0.94 (1.12)	-0.413 (-0.708)	-0.149 (-0.186)	0.530 (0.553)	0.31	0.27	1	0.59	0.36	0.27	0.28	0.29	0.35	0.38
4. I feel good most of the time. 4. मैं ज़्यादातर समय अच्छा महसूस करता/करती हूँ।	3.66 (3.67)	0.92 (1.05)	-0.350 (-0.481)	-0.382 (-0.486)	0.475 (0.496)	0.21	0.21	0.57	1	0.30	0.22	0.31	0.29	0.30	0.36
5. What I do in life is valuable and worthwhile. 5. मैं जीवन में जो कुछ करता/करती हूँ वह मूल्यवान और सार्थक है।	3.77 (3.76)	0.82 (0.97)	-0.679 (-0.570)	0.873 (0.127)	0.405 (0.480)	0.28	0.28	0.31	0.26	1	0.25	0.33	0.32	0.26	0.34
6. I can succeed if I put my mind to it. 6. मैं उस कार्य में सफल हो सकता/सकती हूँ यदि मैं उसमे अपना दिमाग लगा सकूँ।	4.38 (4.34)	0.81 (0.93)	-1.388 (-1.730)	1.906 (3.101)	0.375 (0.472)	0.30	0.33	0.19	0.19	0.22	1	0.18	0.41	0.32	0.26
7. I am achieving most of my goals. 7. मैं अपने अधिकांश लक्ष्यों को प्राप्त कर रहा/रही हूँ।	3.46 (3.78)	0.92 (1.03)	0.067 (-0.635)	-0.665 (-0.107)	0.473 (0.510)	0.31	0.28	0.33	0.33	0.26	0.16	1	0.44	0.37	0.34
8. In most activities I do, I feel energized. 8. मैं अपने द्वारा की जाने वाली अधिकांश गतिविधियों में ऊर्जावान महसूस करता/करती हूँ।	3.72 (3.85)	0.88 (1.04)	-0.390 (-0.641)	-0.142 (-0.200)	0.392 (0.575)	0.33	0.28	0.26	0.27	0.32	0.32	0.36	1	0.40	0.40
9. There are people who appreciate me as a person. 9. मेरे जीवन में ऐसे लोग हैं जो एक व्यक्ति के रूप में मेरी सराहना करते हैं।	3.90 (3.91)	0.96 (1.05)	-0.858 (-1.020)	0.433 (0.628)	0.490 (0.514)	0.21	0.23	0.33	0.30	0.26	0.27	0.28	0.37	1	0.46
10. I feel a sense of belonging in my community. 10. मुझे अपने समुदाय में अपनेपन की अनुभूति होती है।	3.72 (4.01)	0.88 (1.06)	-0.585 (-1.016)	0.233 (0.521)	0.479 (0.549)	0.23	0.21	0.36	0.35	0.28	0.19	0.33	0.34	0.41	1

BIT-E = English version of the Brief Inventory of Thriving; BIT-H = Hindi version of the Brief Inventory of Thriving; Sk = skewness; Ku = kurtosis; ITC = Item-total correlation; numerals in parenthesis indicate values for BIT-H; Column 1–10 indicates inter-item correlations for BIT-E (below diagonal) and BIT-H (above diagonal); all inter-item correlations are significant at 0.001 level.

#### Data analyses strategy

We analyzed the data in multiple steps to meet the study objectives. First, we evaluated the unidimensional structure of the BIT-E and the BIT-H through confirmatory factor analysis (CFA). Unidimensionality pertains to a single factor underlying a set of items on the scale. It is accepted when all the factor loadings are greater than 0.30 and statistically significant, with an acceptable fit to the data^43^. We based our decision to accept the model fit on χ^2^ statistic (with its corresponding *p-value*) and four fit indices: (a) the Comparative Fit Index (CFI), (b) the Tucker-Lewis Index (TLI), (c) the Root Mean Square Error Approximation (RMSEA), with its 90% confidence interval, and (d) the Standardized Root Mean Squared Residuals (SRMR). A non-significant value of χ^2^ indicates that the model is consistent with the data. However, χ^2^ is sensitive to sample size and approaches significance when the sample size is large^44^. Therefore, we also used normed chi-square (χ^2^/*df*) to determine the model fit. The value of χ^2^/*df* smaller than 2 and 3 indicates a good and acceptable fit, respectively^45^. For CFI and TLI, the values at or greater than 0.90 and 0.95 are an acceptable and excellent fit to the data, respectively. For RMSEA and SRMR, the values at or less than 0.08 and 0.05 reflect reasonable and close fit, respectively^46^. Since the data could not meet the assumption of multivariate normality, we used Maximum Likelihood Estimation with Robust Standard Errors (MLM) to evaluate the unidimensional structure of the BIT-E and BIT-H. MLM provides the Satorra-Bentler scaled chi-square (S-Bχ^2^) statistic, which is robust to non-normality^47^. Further, we examined modification indices, with values above 10.0 as indicative of substantial misfit, in conjunction with their theoretical relevance to improving model fitness (Byrne, 2010). As the original language of the BIT is English, the best-fitting CFA model of BIT-E served as the reference model for BIT-H and was replicated to establish the unidimensionality of BIT-H.

The second step involved testing the BIT’s measurement invariance (MI) across language versions, gender, and academic disciplines using multi-group confirmatory factor analysis (MG-CFA). MI involves testing whether the construct being validated measures the same underlying construct with similar meaning to its constituent items across various demographic groups of interest^33^. It includes comparing models across groups in which the factor structure is systematically set for equality constraints generally at four different levels, including configural, metric, scalar, and strict. The first level involves testing the configural invariance model. Being the least constrained model, configural invariance tests whether the different groups have the same factor structure. If the scale achieves configural invariance, it is concluded that factor structure is similar across groups. Subsequently, increasingly restrictive equality constraints are imposed on specific parameters across groups, resulting in nested models that are compared using the χ^2^ difference test^43^. Metric invariance is tested at the second level by constraining factor loadings to test whether factor loadings are equivalent across groups. Meeting metric invariance is essential for comparing predictive relationships across groups^32^. The third level involves testing scalar invariance by constraining factor loadings and item intercepts to assess whether the intercepts of the items are equivalent. For comparing group latent means, a scalar level of equivalence is required^44^. At the fourth level, strict invariance is tested by constraining factor loadings, item intercepts, and residual errors. Meeting strict invariance is essential for comparing group means through the observed raw scores. Each subsequent level of invariance has more constraints than the previous one, resulting in nested models. For example, metric invariance is nested within configural invariance and tested using a χ^2^ difference test^43^. If the χ^2^ difference test results in a non-significant value, measurement invariance is accepted for that level. Violation in the multivariate normality assumption led us to use the Satorra and Bentler scaled chi-square (S-Bχ^2^) difference test to compare the nested models^47^. We also examined the change in CFI (ΔCFI) and RMSEA (ΔRMSEA) values between the nested models^44^. Invariance can be claimed for the nested model if ΔCFI and ΔRMSEA are less than 0.01 and 0.015, respectively. If the model suggested a lack of invariance, we tested for partial invariance on specific parameters lacking invariance across groups. Significant parameters adversely impacting the model fit were identified using the *ccpsyc* package^48^ to test the partial invariance. Then, based on the identified parameters, we freed the constraints from the most significant parameter impacting model fit iteratively until the model met the recommended cut-off for partial invariance^49^. If 80% of items on a scale demonstrate complete measurement invariance, it is inferred that the instrument has structural validity evidence for the target groups and may be used for cross-group comparison^30,33^. Measurement invariance between the English and Hindi versions was tested first at the four levels described, followed by testing invariance across gender and academic discipline.

In the next step, we evaluated the reliability and validity of the BIT. The reliability (pertaining to the consistency) of the BIT was assessed by testing internal consistency, item-total correlation (ITC), and inter-item correlation (IIC). Internal consistency was estimated by the McDonald’s Omega (*ω*) coefficient^50^. We considered *ω* value ≥ 0.70 and ITCs above 0.20, and mean IIC within the range of 0.15 − 0.50 as indicators of adequate reliability^51^. For validity (pertaining to accuracy), convergent/discriminant, and criterion validity were assessed using Pearson’s product-moment correlation coefficient. We determined the convergent/discriminant validity of the BIT- E and BIT- H based on a significant correlation in an expected direction with scales measuring similar constructs (life satisfaction, positive and negative affect, subjective happiness, and student satisfaction). These correlations should be less than 0.85 to determine discriminant validity^52^. For criterion validity, the BIT-H should show a significantly positive correlation with theoretically relevant constructs such as generic skills, academic self-efficacy, student engagement, and perseverance.

The missing values and Pearson’s product-moment correlations were analyzed using IBM SPSS (version 21). CFA and MGCFA were conducted using R (4.2.2) and R Studio (2023.03.0 + 386). The *lavaan* package (0.6–13), with the default marker method, was used for CFA and MGCFA^53^.

## Results

### Unidimensionality assessment

As shown in Table 2, the unidimensional structure of the BIT-E resulted in an almost acceptable fit: S-Bχ^2^(35) = 67.985; *p* = .001; S-Bχ^2^/*df* = 1.94; CFI_scaled_ = 0.902; TLI_scaled_ = 0.873; RMSEA_scaled_ = 0.071, 90% *CI* [0.045-0.096]; SRMR = 0.060. We examined the modification indices to locate the misfit in the model. The highest modification indices suggested freeing the error covariances between items 3 and 4 (Modification indices = 17.46) and between items 1 and 2 (Modification indices = 12.50).

**Table 2:** Model fit indices for the BIT-E and BIT-H.

Sample 1 (*N*_1_ = 224)	S-Bχ^2^ (df)	*p*	S-Bχ^2^ /df	CFI_scaled_	TLI_scaled_	RMSEA_scaled_ [90% CI]	SRMR	Sample 2 (*N*_2_ = 310)	S-Bχ^2^ (df)	*p*	S-Bχ^2^ /df		CFI_scaled_	TLI_scaled_	RMSEA_scaled_ [90% CI]	SRMR
BIT-E	67.985 (35)	0.001	1.94	0.902	0.873	0.071 [0.045-0.096]	0.060	BIT-H	113.230 (35)	0.001		3.23	0.855	0.814	0.101 [0.080–0.122]	0.067
BIT-E_T3~T4_	53.242 (34)	0.019	1.56	0.942	0.923	0.055 [0.023-0.083]	0.054	BIT-H _T3~T4_	68.396 (34)	0.001		2.01	0.936	0.916	0.068 [0.044–0.091]	0.054
**BIT-E**_**T1~T2**_	**45.559 (33)**	**0.072**	**1.38**	**0.963**	**0.949**	**0.045** **[0.000-0.074]**	**0.050**	**BIT-H**_**T1~T2**_	**54.041** **(33)**	**0.012**		**1.63**	**0.961**	**0.947**	**0.054** **[0.025–0.079]**	**0.048**

BIT-E = English version of the Brief Inventory of Thriving; BIT-H = Hindi version of the Brief Inventory of Thriving; BIT-E/H _T3~T4_ = modification indices between item 3 and item 4; BIT-E/H _T1~T2_ = modification indices between item 1 and item 2; S-Bχ^2^ = Satorra-Bentler Chi-Square; *df* = degree of freedom; CFI_scaled_ = robust comparative fit index; TLI_scaled_ = robust Tucker-Lewis index; RMSEA_scaled_ = robust root-mean-square error of approximation; *CI* = confidence interval; SRMR = standardized root-mean-square residual; Bolded = best-fitting model.

Items 3 (“*My life is going well*”) and 4 (“*I feel good most of the time*”) measure *life satisfaction* and *positive affect*, respectively, while items 1 (“*My life has a clear sense of purpose*”) and 2 (“*I am optimistic about my future*”) measure *purpose in life* and *optimism*, respectively. Therefore, the residuals between items 3 and 4 and between items 1 and 2 were freed to correlate due to their theoretical relevance. For clarity, we first tested an intermediate model by freeing the residuals between items 3 and 4, which showed an improved fit (see Table 2). Finally, after adding residuals between 1 and 2, the model demonstrated an excellent model fit: S-Bχ^2^(33) = 45.559; *p* = .072; S-Bχ^2^/*df* = 1.38; CFI_scaled_ = 0.963; TLI_scaled_ = 0.949; RMSEA_scaled_ = 0.045, 90% *CI* [0.000 − 0.074]; SRMR = 0.050.

The final model of the BIT-E (Sample 1) served as the reference model for BIT-H (Sample 2). The unidimensional model with no correlated residuals resulted in a poor model fit for BIT-H: S-Bχ^2^(35) = 113.230; *p* < .001; S-Bχ^2^/*df* = 3.23; CFI_scaled_ = 0.855; TLI_scaled_ = 0.814; RMSEA_scaled_ = 0.101, 90% *CI* [0.080-0.122]; SRMR = 0.067. Then, we correlated the residuals between items 3 and 4 (Modification indices = 63.06) and between items 1 and 2 (Modification indices = 22.96), resulting in an excellent model fit: S-Bχ^2^(33) = 54.041; *p* = .012; S-Bχ^2^/*df* = 1.63; CFI_scaled_ = 0.961; TLI_scaled_ = 0.947; RMSEA_scaled_ = 0.054, 90% *CI* [0.025-0.079]; SRMR = 0.048.

**Fig. 1:**
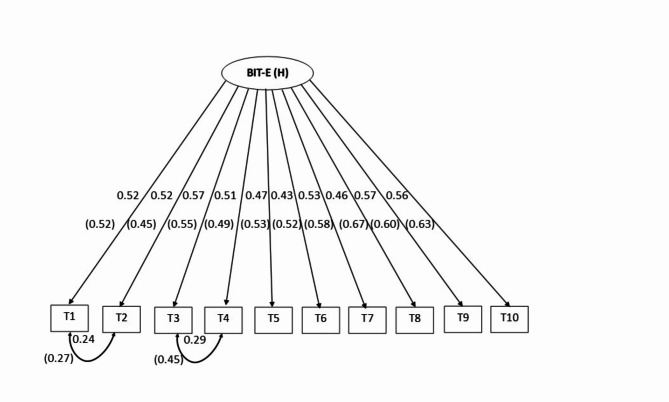
The Unidimensional Model of the BIT-E and BIT-H with Factor Loadings and two pairs of Correlated Residuals.* Note*: BIT-E (H) = English (Hindi) version of the Brief Inventory of Thriving. Values for the Brief Inventory of Thriving (Hindi version) are indicated in parenthesis.

Figure 1 indicates the unidimensional model of the BIT-E and BIT-H; all standardized path coefficients were significant (*p* < .001) and greater than 0.30. Since the unidimensional model for both versions demonstrated an excellent fit to the data, we combined the samples, and as expected, the combined data also resulted in an excellent model fit: S-Bχ^2^(33) = 60.880; *p* = .002; S-Bχ^2^/*df* = 1.84; CFI_scaled_ = 0.968; TLI_scaled_ = 0.956; RMSEA_scaled_ = 0.046, 90% *CI* [0.027-0.064]; SRMR = 0.040. Therefore, in the next step, two groups were defined based on the participants who filled the BIT-E (Sample 1) or BIT-H (Sample 2) to test the measurement invariance between them.

### Measurement invariance between the BIT-E and BIT-H

Table 3 demonstrates the result of measurement invariance between the BIT-E and BIT-H. The configural model demonstrated excellent fit: S-Bχ^2^(66) = 100.277; *p* = .004; S-Bχ^2^/*df* = 1.51; CFI_scaled_ = 0.962; TLI_scaled_ = 0.948; RMSEA_scaled_ = 0.050, 90% *CI* [0.029-0.069]; SRMR = 0.045. Then, we tested the metric invariance by adding equality constraints on the factor loadings across both versions. The comparison between the metric (model 2) and configural (model 1) model revealed a nonsignificant decrement in the model fit, *T*_*d*_ (9) = 5.081, *p* = .827. ΔCFI_scaled_ and ΔRMSEA_scaled_ were also within the acceptable range (see Table 3). Then, we tested scalar invariance (model 3) by adding equality constraints on the intercepts and compared it with the metric model (model 2), which significantly decreased the model fit: *T*_*d*_ (9) = 34.058, *p* < .001. ΔCFI_scaled_ also exceeded the acceptable range, whereas ΔRMSEA_scaled_ was within the range. We next examined the parameters that caused potential misfits in the model to test partial scalar invariance using the *ccpsyc* package^48^. Based on the results, we freed intercepts for item 7. Freeing of item 7 (model 3a) still resulted in a significant decrement in model fit when compared with the metric model (model 2): *T*_*d*_ (8) = 25.188, *p* < .001. ΔCFI_scaled_ also exceeded the acceptable range. The same process was reapplied, and this time we freed intercepts for item 10 (model 3b), which again resulted in a significant decrement in model fit when compared with model 2: *T*_*d*_ (7) = 15.928, *p* = .025. However, we decided to accept model 3b, as ΔCFI_scaled_ and ΔRMSEA_scaled_ both were within the range, indicating partial scalar invariance. The intercepts for items 7 and 10 were higher for the Hindi version than for the English version (for item 7, *M*_BIT−E_ = 3.46 vs. *M*_BIT−H_ = 3.78: for item 10, *M*_BIT−E_ = 3.72 vs. *M*_BIT−H_ = 4.01), indicating that the means on these single items are not directly comparable, but the other variables are directly comparable. This implies that the factor structure is comparable across the BIT-E and the BIT-H, but care should be taken in comparing mean level responses.

**Table 3:** Measurement invariance of the BIT across language versions, gender, and academic disciplines.

	Model	S-Bχ^2^ (df)	*P*	S-Bχ^2^ /df	CFI_scaled_	TLI_scaled_	RMSEA_scaled_ [90% CI]	SRMR	Model Comparison	T_d_	Δdf	*p*	ΔCFI_scaled_	ΔRMSEA_scaled_
BIT-E (*N* = 224) Vs. BIT-H (*N* = 310)	1. Configural	100.277 (66)	0.004	1.51	0.962	0.948	0.050 [0.029-0.069]	0.045						
	2. Metric	105.647 (75)	0.011	1.40	0.966	0.959	0.044 [0.022-0.063]	0.050	1 and 2	5.081	9	0.827	0.004	0.006
	3. Scalar	133.628 (84)	0.001	1.59	0.947	0.943	0.053 [0.035-0.069]	0.056	2 and 3	34.058	9	0.001	0.019	0.009
	3 a. Scalar_T7~1_	126.923 (83)	0.001	1.52	0.953	0.949	0.050 [0.031-0.067]	0.055	2 and 3a	25.188	8	0.001	0.013	0.006
	3 b. Scalar_T10~1_	119.980 (82)	0.004	1.46	0.959	0.955	0.047 [0.027-0.064]	0.053	2 and 3b	15.928	7	0.025	0.007	0.003
	4. Strict	166.341 (92)	0.001	1.80	0.921	0.923	0.061 [0.046-0.076]	0.074	3b and 4	52.173	10	0.001	0.038	0.014
Boys (*N* = 279) Vs. Girls (*N* = 255)	1. Configural	90.711 (66)	0.024	1.37	0.972	0.962	0.043 [0.017-0.064]	0.042						
	2. Metric	113.279 (75)	0.003	1.51	0.957	0.948	0.050 [0.030-0.069]	0.065	1 and 2	22.046	9	0.008	0.015	0.007
	2a. Metric = ~ _T6_	100.441 (74)	0.022	1.35	0.970	0.964	0.042 [0.017-0.062]	0.052	1 and 2a	9.740	8	0.283	0.002	0.001
	3. Scalar	117.401 (83)	0.008	1.41	0.963	0.960	0.045 [0.024-0.062]	0.055	2a and 3	18.84	9	0.026	0.007	0.003
	4. Strict	124.172 (93)	0.017	1.33	0.966	0.967	0.040 [0.018-0.058]	0.058	3 and 4	6.988	10	0.726	0.003	0.005
Science (*N* = 358) Vs. Commerce (*N* = 145)	1. Configural	99.437 (66)	0.005	1.50	0.959	0.944	0.052 [0.029-0.072]	0.048						
	2. Metric	112.667 (75)	0.003	1.50	0.955	0.946	0.051 [0.030-0.070]	0.056	1 and 2	13.182	9	0.154	0.004	0.001
	3. Scalar	131.912 (84)	0.001	1.57	0.944	0.941	0.054 [0.035-0.071]	0.060	2 and 3	21.614	9	0.010	0.011	0.003
	4. Strict	142.751 (94)	0.001	1.51	0.944	0.946	0.051 [0.033-0.067]	0.060	3 and 4	10.695	10	0.381	0.001	0.003

BIT-E = English version of the Brief Inventory of Thriving; BIT-H = Hindi version of the Brief Inventory of Thriving; S-Bχ^2^ = Satorra-Bentler Chi-Square; *df* = degree of freedom; CFI_scaled_ = robust comparative fit index; TLI_scaled_ = robust Tucker-Lewis index; RMSEA_scaled_ = robust root-mean-square error of approximation; *CI* = confidence interval; SRMR = standardized root-mean-square residual; T_7 ~ 1_ = freeing intercept for item 7; T_10 ~ 1_ = freeing intercept for item 10; =~T_6_ = freeing factor loading for item 6; T_*d*_ = scaled difference test statistic; Δ = difference.

### Measurement invariance across gender

We next tested for invariance across gender, using the combined dataset. Table 3 demonstrates the results. The configural model resulted in an excellent fit: S-Bχ^2^ (66) = 90.711; *p* = .024; S-Bχ^2^/*df* = 1.37; CFI_scaled_ = 0.972; TLI_scaled_ = 0.962; RMSEA_scaled_ = 0.043, 90% *CI* [0.017-0.064]; SRMR = 0.042. The comparison between the metric (model 2) and configural model (model 1) revealed a significant decrement in the model fit: *T*_*d*_ (9) = 22.046, *p* = .008. ΔCFI_scaled_ also exceeded the acceptable range, while ΔRMSEA_scaled_ was within the range. Item 6 caused the major misfit in the model, as indicated by modification indices. We released item 6 (model 2a) and compared it with model 1, which resulted in a non-significant decrement in the model fit: *T*_*d*_ (8) = 9.740, *p* = .283. Further, we added equality constraints on intercept to test the scalar invariance (model 3). We then compared it with the metric model (model 2a), which indicated a significant decrement in the model fit: *T*_*d*_ (9) = 18.84, *p* = .026. However, both ΔCFI_scaled_ and ΔRMSEA_scaled_ were within the range, so we retained model 3. In the last step, we added constraints on the residuals to test the strict invariance (model 4) and compared it with the scalar invariance (model 3). It resulted in a non-significant decrement in the model fit: *T*_*d*_ (10) = 6.988, *p* = .726, supporting strict invariance. According to Dimitrov, invariance can be claimed if the number of non-invariant items is small^33^. Since only 1 item indicated non-invariance, we conducted an independent sample t-test, which revealed no significant difference between the well-being scores of boys and girls: *t* (532) = 0.070, *p* = .944. Girls scored (*M =* 38.80) slightly higher than boys (*M* = 38.76).

### Measurement invariance across academic disciplines

We next tested the MI across academic disciplines (science, commerce, and the humanities). The result indicated a convergence issue as the number of participants in the humanities was less than in commerce and science. Therefore, we excluded the participants from the humanities to test MI between Science and Commerce disciplines. Table 3 indicates the results of measurement invariance between Science and Commerce disciplines. The configural model resulted in an excellent fit: S-Bχ^2^(66) = 99.437; *p* = .005; S-Bχ^2^/*df* = 1.50; CFI_scaled_ = 0.959; TLI_scaled_ = 0.944; RMSEA_scaled_ = 0.052, 90% *CI* [0.029-0.072]; SRMR = 0.048. The comparison between the metric (model 2) and configural (model 1) model revealed a non-significant decrement in the model fit: *T*_*d*_ (9) = 13.182, *p* = .154. Then, a comparison between the scalar (model 3) and metric model (model 2) indicated a significant decrement in the model fit: *T*_*d*_ (9) = 21.614, *p* = .010. However, we retained the scalar model as ΔCFI_scaled_ and ΔRMSEA_scaled_ were within the recommended limit. Further, we also accepted the strict invariance model as there was no significant decrement between the strict and scalar model: *T*_*d*_ (10) = 10.695, *p* = .381.

### Internal consistency, item-total, and inter-item correlation

The values of McDonald’s omega (*ω*) were above 0.70 for both versions (ω_BIT−E_ = 0.791; ω_BIT−H_ = 0.826), indicating internal consistency. The ITC for the BIT-E ranged from 0.375 to 0.530 (*M* = 0.459, *SD* = 0.050), whereas it ranged from 0.445 to 0.575 (*M* = 0.510, *SD* = 0.040) for the BIT-H. For BIT-E, the mean IIC was 0.272, while it was 0.320 for BIT-H. These results indicate that both versions have adequate reliability (see Table 1).

### Convergent/discriminant and criterion validity

Table 4 demonstrates the descriptive statistics and correlations among variables to determine the convergent/discriminant and criterion validity of the BIT-E and BIT-H. As expected, the BIT-E demonstrated a significantly positive correlation with life satisfaction (*r﻿* = .28, *p* < .001), positive affect (*r* = .19, *p* < .001), subjective happiness (*r* = .29, *p* < .001), student satisfaction (*r* = .32, *p* < .001), and negatively related to negative affect (*r* = -.22, *p* < .001) suggesting convergent validity of the scale. The correlation between the BIT-H and student satisfaction (*r* = .62, *p* < .001) suggested the convergent validity of the BIT-H. Likewise, these correlations were not too high (< 0.85), providing evidence for discriminant validity^52^. Further, the correlations between the BIT-H with generic skills (*r* = .48, *p* < .001), academic self-efficacy (*r* = .54, *p* < .001), student engagement (*r* = .36, *p* < .001), and perseverance (*r* = .49, *p* < .001) supported the criterion validity of the scale.

**Table 4:** Descriptive statistics and correlation coefficients for BIT-E and BIT-H.

Sample 1 (*N* = 224)	M	SD	1	2	3	4	5	6	Sample 2 (*N* = 310)	M	SD	1	2	3	4	5	6
1. BIT-E	38.07	5.15	(0.791)						1. BIT-H	39.10	9.69	(0.826)					
2. LS	16.07	3.66	0.28^***^	(0.716)					2. GS	32.10	7.10	0.48^***^	(0.868)				
3. PA	15.49	2.82	0.19^***^	0.17^*^	(0.722)				3. ASE	38.50	7.17	0.54^***^	0.67^***^	(0.830)			
4. NA	8.98	3.23	-0.22^***^	-0.30^***^	-0.21^**^	(0.755)			4. SE	18.84	5.46	0.36^***^	0.49^***^	0.53^***^	(0.818)		
5. SH	19.70	3.76	0.29^***^	0.20^**^	0.31^***^	-0.35^***^	(0.704)		5. SP	21.88	4.65	0.49^***^	0.59^***^	0.65^***^	0.53^***^	(0.835)	
6. SS	36.61	5.27	0.32^***^	0.21^**^	0.12	-0.25^***^	0.17^**^	(0.710)	6. SS	37.78	6.82	0.62^***^	0.64^***^	0.62^***^	0.41^***^	0.43^***^	(0.786)

BIT-E = English version of the Brief Inventory of Thriving; LS = Life Satisfaction; PA = Positive Affect; NA = Negative Affect; SH = Subjective Happiness; SS = Student Satisfaction; BIT-H = Hindi version of the Brief Inventory of Thriving; GS = Generic Skills; ASE = Academic Self-efficacy; SE = Student Engagement; SP = Student Perseverance; Values in parentheses indicate the internal consistency reliability of the scales, which was measured using McDonald’s Omega coefficient.

^*^p < .05, ^**^p < .01, ^***^p < .001.

## Discussion

The growing focus on adolescents’ well-being makes it crucial to have reliable and valid tools for assessing their well-being. Measures need to capture well-being directly, be as brief as possible, in the right language for participants, useful across WEIRD and non-WEIRD populations, and validated for use with young people. The Brief Inventory of Thriving (BIT) is a promising measure, as it captures 10 dimensions of well-being, is short and easy to administer, has been translated to other languages, used with WEIRD and non-WEIRD cultures, and has demonstrated good psychometric properties when used with adults. Here we add a Hindi version of the measure and establish the psychometric properties of both the BIT-E and BIT-H for use with Indian adolescents. We used the rigorous methodological approach given by Sousa and Rojjanasrirat to develop the BIT-E’s Hindi-translated version^31^. In this context, the present study provides the first evidence for the psychometric properties of the BIT in Hindi, which is spoken by a vast population of India.

We first tested the unidimensional structure of both BIT-E and BIT-H. We next tested the measurement invariance of the BIT across the two language versions, gender, and academic disciplines. Finally, we assessed the reliability and validity of both versions of the measure. We hypothesized that both versions of the BIT would support their unidimensional structure among Indian adolescents. Our initial analyses indicated sub-optimal fit, which could be fixed by modifying the factor structure to allow the residuals of 4 items to correlate. Including modification indices in the model is controversial^54^. However, previous studies that tested the factor structure of the BIT with adult samples also included modification indices^24,29^. Therefore, we added modification indices in the model by correlating residuals between the items. The first pair of items that required modification was between item 3 (life satisfaction) and item 4 (positive affect). The second pair of items that were identified were item 1 (purpose in life) and item 2 (optimism about the future). These modifications in the model resulted in an excellent fit. The best-fitting CFA model of the BIT-E served as the reference model for BIT-H, which also resulted in an excellent model fit. All the BIT-E and BIT-H items loaded significantly on their unidimensional factor. Overall, these results and the combined sample results supported our first hypothesis. The results also inform the proper factorial model that should be used with the measures, which allows some residual items to be correlated.

Second, we expected measurement invariance across the language versions, gender, and academic discipline. For language invariance, the factor structure and the factor loadings were identical for both versions, implying that the participants conceptualized the thriving construct similarly, and the BIT’s items functioned similarly, whether presented in English or Hindi. These results support that the Hindi version of the BIT is equivalent to its English version. However, the results from scalar invariance indicated that intercepts for item 7 *(“I am achieving most of my goals”)* and item 10 *(“I feel a sense of belonging in my community”)* were not equivalent, with higher scores observed in the BIT-H sample. This suggests that while factor level results are comparable across language versions, mean scores are not directly comparable. This may be attributed to differences in contextual and educational experiences, which influence how accomplishment and sense of belonging are perceived across the two language groups. Releasing the constraints from these two items resulted in partial scalar invariance, supporting our second hypothesis. These results suggest that norm values need to be established for each version of the measure, at least with Indian participants. The descriptives (Table 1) here provide mean values and standard deviations that other studies might compare to, for both the BIT-E and the BIT-H.

The measurement invariance test across genders achieved strict measurement invariance for most items, except for item 6 (*“I can succeed if I put my mind to it”*), which demonstrated differential factor loadings for boys and girls. Thus, the BIT achieved partial measurement invariance rather than full measurement invariance across gender. Despite this, the fact that 90% of the items demonstrated invariance across gender suggests that boys and girls conceptualize the construct of thriving similarly, attribute the same meaning to its constituent items, and have equivalent levels (intercepts) of the underlying construct, with minimal concern regarding differential measurement error^55^. These findings align with the previous study^30^ and support our second hypothesis. Given the mixed evidence on gender differences in well-being^56^, we conducted an independent sample *t-*test on the well-being scores of boys and girls, which revealed no significant differences. Our result aligns with the findings from^57^, who also reported similar levels of psychological well-being among boys and girls.

Additionally, we explored the measurement invariance of the BIT between science and commerce disciplines, which demonstrated strict invariance. Achieving full scalar and strict invariance enhances the validity and reliability of cross-discipline comparisons, allowing researchers to confidently interpret and generalize findings across different academic disciplines groups (Commerce and Science). These results suggest that the measure is comparable across key demographic variables, such that analyses do not need to be adjusted for academic discipline. We do note that we only compared science and commerce as academic disciplines, due to the low number of participants in the humanities. Further testing across disciplines might be useful for ascertaining well-being scores within specific subjects, moving beyond overall well-being at school.

Finally, we expected to find good reliability and validity of the BIT-E and BIT-H. As expected, McDonald’s Omega, item-total, and inter-item correlations demonstrated good internal consistency for BIT-E and BIT-H. Further, BIT-E showed the anticipated pattern of correlations, with significant positive correlations with measures of well-being and negative correlations with negative affect. This supports the convergent and discriminant validity of BIT-E. The positive correlations observed between the BIT-E and measures of well-being such as life satisfaction, positive affect, subjective happiness, and student satisfaction suggest that adolescents who reported higher levels of thriving on BIT-E tended to report higher levels of these positive indicators of well-being. This indicates that BIT-E effectively captures the construct of thriving as it relates to positive psychological functioning. Additionally, the negative correlation between BIT-E and negative affect suggests that individuals who report higher levels of thriving tend to report lower levels of negative emotions, further supporting the validity of BIT-E in distinguishing thriving from negative affective states. Further, the positive correlation with generic skills in school, academic self-efficacy, student engagement, and perseverance, suggests that BIT-H is related to adolescents’ academic functioning. The small to medium effect sizes of these correlations suggest that while the relationships are significant, they are not overly strong, indicating that thriving is related to, but distinct from, other constructs such as student satisfaction and academic engagement. Overall, these findings provide support for the validity of both BIT-E and BIT-H, indicating that they effectively measure the construct of thriving across different linguistic contexts and are valuable tools for assessing well-being among adolescents.

### Limitations and future directions

The present study has several limitations. First, the measures we used in this study were all self-reported. Future studies might include interview-based assessment and cross-informant approaches to incorporate multi-trait-multi-method analyses for assessing construct validity and considering method variance. Second, the criterion measures used in the study are related to school-related outcomes. Future studies might use health-related measures such as those used in the original study^20^. Third, the study involved cross-sectional data, such that test-retest reliability and measurement invariance over time could not be tested. Future studies might use a longitudinal design to assess the functioning of the BIT-E and BIT-H across time among adolescents. Fourth, the survey questions were presented in the same order for all participants, and there could be order effects at work. Future studies might randomly present items and test for order effects, both within and across well-being measures. Fifth, the sample size was limited to several hundred participants per survey, which is inadequate for establishing norm values. The partial scalar invariance found between the BIT-E and BIT-H indicated that mean values are not directly comparable. Future research should collect larger samples with both versions to establish normed responses for comparison. Finally, all the participants in the present study were recruited from the Hindi-speaking region of India. This limits the generalizability of findings to a single culture, and results may not generalize across other cultures, and care should be taken when comparing means across cultures and languages. Future studies might focus on testing the measurement invariance of the various versions of the BIT for adolescents from different cultural backgrounds to test the external validity of the two versions of the BIT measure.

## Conclusion

The findings of our study have the potential to inform policies, interventions, and applied settings that target enhancing the well-being of adolescents, thereby contributing to their positive development. With the robust psychometric properties of BIT-E and BIT-H among adolescents, the findings have implications for the researchers and mental health professionals who may use both BIT-E and BIT-H in developmental and educational programs to assess and improve their well-being, depending on their needs and the context. These practical applications hold promise for informed decision-making, the design of targeted interventions, and the development of policies that foster positive youth development, ultimately contributing to healthier and thriving adolescent populations.

## Data Availability

The data related to the present study is available with the corresponding author and can be made available on reasonable request.
